# Carbon Nanosheets Grown via RF-PECVD on Graphite Films and Thermal Properties of Graphite Film/Aluminum Composites

**DOI:** 10.3390/nano15100773

**Published:** 2025-05-21

**Authors:** Yifu Ma, Jinrui Qian, Ping Zhu, Junyao Ding, Kai Sun, Huasong Gou, Rustam Abirov, Qiang Zhang

**Affiliations:** 1 School of Materials Science and Engineering, Harbin Institute of Technology, Harbin 150001, China; 23b909042@stu.hit.edu.cn (Y.M.); d202310146@xs.ustb.edu.cn (J.Q.); 21b909014@stu.hit.edu.cn (P.Z.) 22s109188@stu.hit.edu.cn (J.D.); 20b909018@stu.hit.edu.cn (K.S.); ghs@hit.edu.cn (H.G.); 2 Institute of Mechanics and Seismic Stability of Structures Named after M. T. Urazbaev, Tashkent 100125, Uzbekistan; instmech@academy.uz; 3 State Key Laboratory of Precision Welding & Joining of Materials and Structures, Harbin Institute of Technology, Harbin 150001, China

**Keywords:** RF-PECVD, carbon nanosheets, graphite film/aluminum composites, thermal conductivity

## Abstract

In this study, carbon nanosheets were deposited on the surface of graphite films for surface modification using radio frequency plasma enhanced chemical vapor deposition (RF-PECVD) method. The effects of catalyst addition and concentration, growth gas flow rate, and hydrogen plasma pretreatment on the size, morphology, and density of the deposited carbon nanosheets were investigated. These factors influence the deposition results by affecting the nucleation and growth processes of the carbon nanosheets, while the growth process affects their size. The surface morphology and distribution of the carbon nanosheets were characterized using scanning electron microscopy (SEM). Graphite film/aluminum composites were prepared using graphite films modified under different process conditions as reinforcements. The composite prepared with graphite films modified without catalysts showed significant improvement in thermal conductivity, achieving an xy-direction thermal conductivity of 705 W/(m·K) and a z-direction thermal conductivity of 14.8 W/(m·K), both of which are higher than those of unmodified graphite film/aluminum composites. X-ray diffraction (XRD) analysis was conducted to identify the phase composition of the resulting composites and confirm the structural integrity of the reinforcement after processing.

## 1. Introduction

With the advancement of the electronic information industry, the rapid development of data science and artificial intelligence disciplines has led to increasingly higher performance requirements for electronic devices. As a result, electronics are gradually becoming smaller and more integrated [1]. As predicted by Moore’s law, the number of transistors that can be accommodated per unit area on an integrated circuit doubles every 18 months. As transistor density rapidly increases, the thermal design power of integrated circuits rises as well, ultimately resulting in an exponential increase in the heat generated by circuits [2]. The heat dissipation problem of integrated circuits has become a bottleneck restricting the development of the electronic information industry. In the thermal management of electronic packaging, thermal management materials must rapidly equalize temperatures and dissipate heat efficiently to reduce both the overall and local temperatures of the circuits. These materials must not only have a low coefficient of thermal expansion that matches circuits made from silicon, germanium, or gallium nitride, ensuring proper adhesion, but also possess extremely high thermal conductivity to effectively transfer heat [3].

Metal matrix composites offer design flexibility by combining reinforcements with high thermal conductivity with a metal matrix that exhibits strong machinability and toughness, enabling the customization of thermal physical properties of composites to suit various application environments [4]. Carbon materials such as diamond, graphene, and carbon nanotubes, with their low density, low thermal expansion, and high thermal conductivity, serve as excellent heat dissipation carriers and ideal high thermal conductivity reinforcements [5]. The most commonly used metal matrices in high thermal conductivity composites are copper and aluminum, with aluminum matrix composites offering the added benefits of low density and high specific strength [6]. Although carbon nanotubes and graphene possess the highest thermal conductivity, their large surface area makes them prone to agglomeration during the preparation process. This agglomeration leads to low density in the composite material, resulting in poor thermal conductivity. To prevent agglomeration, the addition of carbon nanotubes and graphene is typically kept below 10%, which causes the thermal conductivity of the composite material to fall significantly short of expectations [7]. In addition to nanocarbon materials and diamond, graphite in carbon materials also exhibits extremely high thermal properties [5]. Depending on the orientation of the graphite crystals, it can be categorized into isotropic and anisotropic graphite. Anisotropic graphite has a layered structure, with extremely high thermal conductivity in the in-plane direction, while the thermal conductivity between layers is relatively low, making it an ideal two-dimensional heat dissipation material. Flake graphite [8] and graphite film [9] are typical examples of anisotropic graphite. High thermal conductivity graphite/aluminum composites inherit the anisotropy of graphite, meaning that orientation has a significant impact on the thermal conductivity of the composite material. Composites with better orientation exhibit higher thermal conductivity [10]. Li et al. adjusted the angle of graphite flakes in the composites by controlling the diameter of the aluminum powder [11]. As the angle decreased from 7.3 to 4.4 degrees, the thermal conductivity increased from 473 to 555 W/(m·K). Compared to flake graphite, graphite films have orientations that are easier to control, making it relatively simpler to achieve near 100% alignment during preparation [12]. During preparation, graphite/aluminum composites face challenges similar to those encountered with other carbon-aluminum composites, such as poor interfacial wettability and weak interfacial bonding [13]. Common optimization methods include matrix alloying and surface modification of the reinforcements [14].

Among the alloying elements in aluminum alloys, the addition of silicon (Si) can effectively enhance the wettability of aluminum alloys on graphite surfaces. In composites with a volume fraction of 30%, the addition of Si significantly reduces interfacial voids and prevents the formation of Al_4_C_3_ [15]. As a result, the thermal conductivity increases from 340 W/(m·K) to 460 W/(m·K) compared to composites without Si [16]. The main surface modification layers are metals and carbides. Appropriate coatings could enhance the interfacial bonding strength. For example, co-plating copper and nickel on the surface of flake graphite can increase the thermal conductivity of the composites from 250 W/(m·K) to 474 W/(m·K) [17]. Coating the surface of graphite films with TiC can effectively improve the interfacial bonding strength of the composite, leading to an increase in thermal conductivity from 440 W/(m·K) to 499 W/(m·K) [18]. Although metal and carbide coatings could improve interfacial bonding of composites, there is a significant difference in thermal conductivity between the coating materials and the graphite film or aluminum matrix, leading to higher interfacial thermal resistance. Compared to metal or carbide coatings, in situ grown nanocarbon material coatings offer higher thermal conductivity. Additionally, in situ growth can effectively reduce the agglomeration of nanocarbon materials during the composite preparation process, allowing for the full utilization of their high thermal conductivity properties. This approach holds considerable potential for enhancing composite performance.

Carbon nanosheets (CNSs) are two-dimensional carbon nanomaterials composed of several to dozens of stacked graphene layers. They typically exhibit a sheet-like morphology with open edges and relatively large lateral dimensions [19,20]. Vertically aligned carbon nanosheets can be deposited on a variety of substrates using plasma-enhanced chemical vapor deposition (PECVD). This technique offers a broad processing window, and the morphology, size, and distribution of the deposited nanosheets are highly sensitive to deposition parameters such as chamber pressure, gas flow rate, substrate temperature, bias voltage, RF power, and the presence or type of catalyst. These characteristics make PECVD particularly suitable for interface modification applications. In addition to experimental investigations of the deposited structures, density functional theory (DFT) modeling and simulations have been employed to gain insights into the synthesis and growth mechanisms of such carbon nanostructures, thereby supporting the optimization of deposition processes [21,22].

In this study, vertically aligned carbon nanosheet arrays were in situ deposited on the surface of graphite films using by plasma enhanced chemical vapor deposition (RF-PECVD) method. The morphology and size variations of the carbon nanosheets were investigated under different conditions, including varying the ratio of precursor gases, catalyst concentration, and plasma etching parameters. A preliminary analysis of the underlying mechanisms was also conducted. Graphite film/aluminum (GF/Al) composites were then prepared using representative process conditions, and the effect of incorporating carbon nanosheet arrays on the thermal conductivity of the composites was examined.

## 2. Materials and Methods

### 2.1. Raw Material

The graphite films used in the experiment were artificial graphite films obtained from polyimide film through high-temperature graphitization, with an average thickness of 20 μm. (Provided by Qingdao Dongke Graphite Co., Ltd., Qingdao, China). The aluminum foil used in the experiment had an average thickness of 23 μm (provided by Jinan Longshan Aluminum Industry Co., Ltd., Jinan, China).

### 2.2. The Growth of Carbon Nanosheets with RF-PECVD

Using the RF-PECVD method, carbon nanosheets were deposited on the surface of graphite films, with methane serving as the precursor to provide the carbon source (CH_4_) and hydrogen (H_2_) as the carrier gas. The entire deposition process consists of three main steps: graphite film pretreatment, catalyst reduction and etching, and carbon nanosheet growth. The parameters for each of these steps were adjusted accordingly. Below is a detailed description of the processes and the chosen parameters:

The first step is the graphite film pretreatment process. The graphite film was soaked in an acetone solution and subjected to ultrasonic treatment for 30 min. After soaking for 12 h, the film was placed in a vacuum drying oven and dried at 60 °C until it achieved a constant weight. The catalyst attachment was performed using a solution immersion method. Nickel nitrate hexahydrate was used as the solute, and anhydrous ethanol was used as the solvent in catalyst concentrations of 0.07 mol/L, 0.10 mol/L, and 0.14 mol/L. The graphite film was immersed in the solution for 24 h, then removed, spread flat in a glass dish, and dried in a vacuum drying oven at 50 °C until it achieved a constant weight.

The second step is the catalyst reduction and etching process. The catalyst particles attached via the solution immersion method need to be reduced to metal particles during the chemical vapor deposition (CVD) heating process by introducing hydrogen.

After reaching the deposition temperature during the CVD process, the growth did not commence immediately. Instead, hydrogen plasma generated by RF was used to etch the catalyst particles on the surface of the graphite film to control their size. The etching was performed at a temperature of 650 °C with a gas flow rate of 15 sccm, and the etching time used was 10 min.

The final step is PECVD growth process. The PECVD growth process involves several parameters, including the heating time, heating rate, gas flow rates, and plasma RF power frequency. The finalized PECVD growth process for the surface carbon nanostructures was as follows: heating time of 30 min, growth temperature of 650 °C, growth time of 30 min, gas flow rates of CH_4_:H_2_ at ratios of 12:3, 16:4, and 20:5 (sccm), and RF power frequency of 300 kHz.

### 2.3. Preparation of the Composites

**Figure 1:**
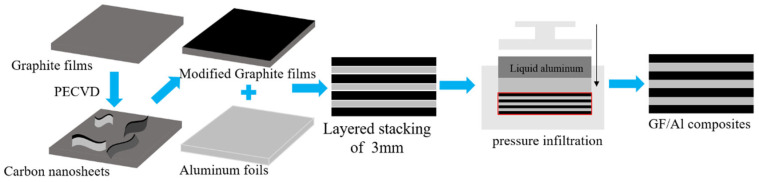
Schematic illustration of GF/Al composites.

The composite was then prepared as follows. To ensure proper infiltration, infiltration channels were pre-fabricated within the graphite films by adding aluminum foils. The graphite films and aluminum foils were alternately stacked layer by layer, with a total of 50 graphite films and 52 aluminum foils stacked to form a preform approximately 3 mm thick. The composite material was then prepared using the pressure infiltration method with 6063 aluminum solution. The schematic illustration is shown in Figure 1.

To eliminate the negative effects caused by an overly thick oxide film, the aluminum foil needed to be cleaned sequentially with sodium hydroxide solution and dilute nitric acid, followed by rinsing with deionized water before use.

### 2.4. Material Characterization

X-ray diffraction (XRD) was used to characterize the graphitization degree of the original graphite film. According to the XRD results, the graphitization degree of the graphite film used was 96.7%. Scanning electron microscopy (SEM) was employed to observe the microstructure of the graphite film before and after modification, as well as that of the composites. Some wrinkles were clearly visible on the surface of the original graphite film. Raman spectroscopy was used to characterize the surface defect density of the graphite film before and after surface modification. The excitation wavelength used was 532 nm. The unmodified graphite film exhibited a smooth region with no noticeable D peak, while the wrinkled region showed the *I*_D_/*I*_G_ ratio of 0.16 (±0.04). Laser flash method was used to measure the thermal diffusion coefficient of both the composites and the graphite film. The thermal conductivity of the composites was calculated based on the formula *λ* = *α*·*ρ*·*C*_p_, where *λ* is the thermal conductivity, α is the thermal diffusion coefficient, and *C*_p_ is the specific heat capacity, which is determined by the rule of mixture.

## 3. Results and Discussion

### 3.1. Growth Factors of Carbon Nanosheets

The effects of catalyst presence and concentration, gas composition, and hydrogen plasma pretreatment on the size and distribution of the grown carbon nanosheets are analyzed in the next section. The size and distribution of carbon nanosheets under different process conditions are shown in Table 1.

#### 3.1.1. Ni Catalyst Concentrations

Since transition metal particles (such as Ni and Fe) have a high carbon solubility and can effectively absorb active carbon species from the plasma, adding catalysts during the deposition of carbon nanosheets has a significant impact on the structure of carbon nanosheets. Compared to the catalyst-free condition, using Ni catalysts effectively increases the density of the carbon nanosheets. Under process conditions without hydrogen plasma etching and with a methane-to-hydrogen flow rate ratio of 16:4, the effects of the Ni catalyst on the size and distribution of the carbon nanosheets were investigated.

**Figure 2:**
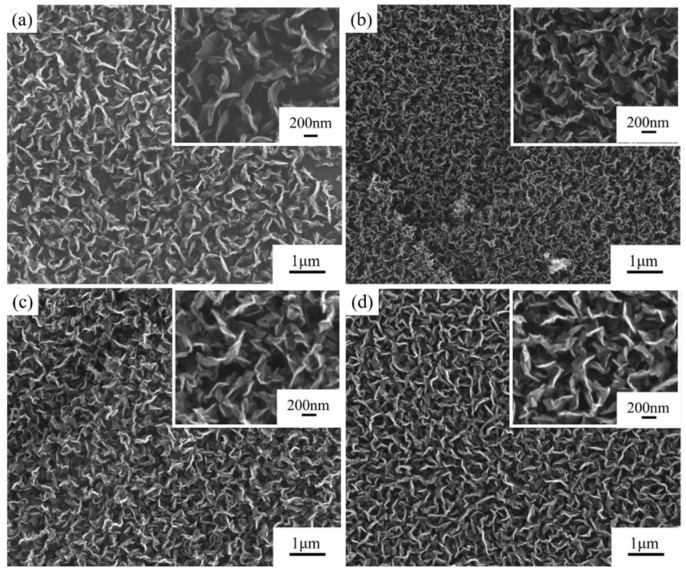
Comparison of structure and properties of carbon nanosheet-modified graphite film prepared with different Ni catalyst concentrations. SEM images of modified graphite film prepared (**a**) without Ni catalyst; and with Ni catalyst concentration of (**b**) 0.07 mol/L; (**c**) 0.10 mol/L; (**d**) 0.14 mol/L.

**Figure 3:**
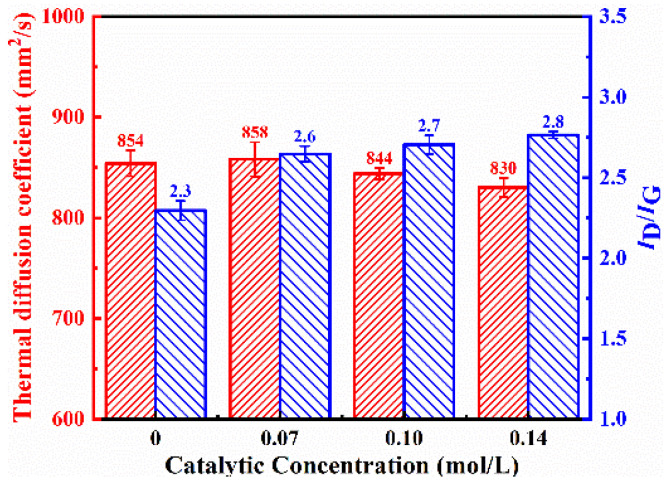
Thermal diffusion coefficient and *I*_D_*/I*_G_ ratio obtained from Raman spectroscopy of graphite film prepared with different Ni catalyst concentrations.

The surface structures of graphite films both without and with the addition of catalysts are shown in Figure 2a,b. The addition of the Ni catalysts significantly increased the density of carbon nanosheets, and there was a slight improvement in the thermal diffusion coefficient, which increased from 854.2 mm^2^/s to 858.3 mm^2^/s. However, the defect density of the carbon nanosheets on the modified graphite film surface also increased, with the *I*_D_/*I*_G_ ratio obtained from Raman spectroscopy results rising from 2.3 to 2.6, which is shown in Figure 3.

The effects of different Ni catalyst concentrations on the modification results are shown in Figure 2b–d. As the catalyst concentration increased, the density of carbon nanosheets did not change significantly, but the flatness and length of the carbon nanosheets increased, although the overall variation was limited. The catalyst’s stronger ability to absorb active carbon species led to the carbon nanosheets growing more preferentially along the sheet direction, rather than nucleating at multiple sites. With increasing catalyst concentration, the thermal diffusion coefficient of the modified graphite film decreased, while the defect density increased.

#### 3.1.2. Gas Flow Rates of CH_4_:H_2_

**Figure 4:**
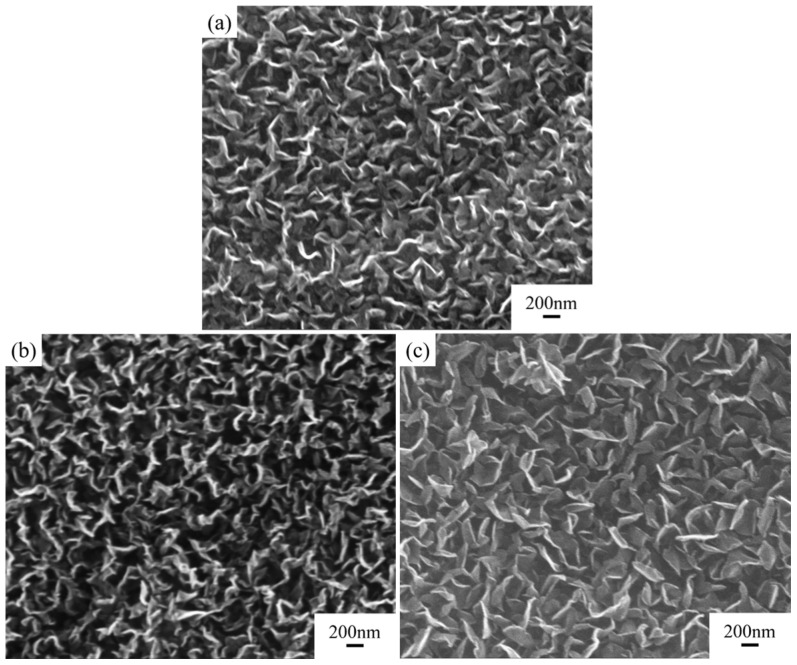
Comparison of structure and properties of carbon nanosheet-modified graphite film pretreated under different grown gas flow rates. SEM images of modified graphite film prepared with CH_4_:H_2_ gas flow rate of (**a**) 12:3 sccm, (**b**) 16:4 sccm, and (**c**) 20:5 sccm.

**Figure 5:**
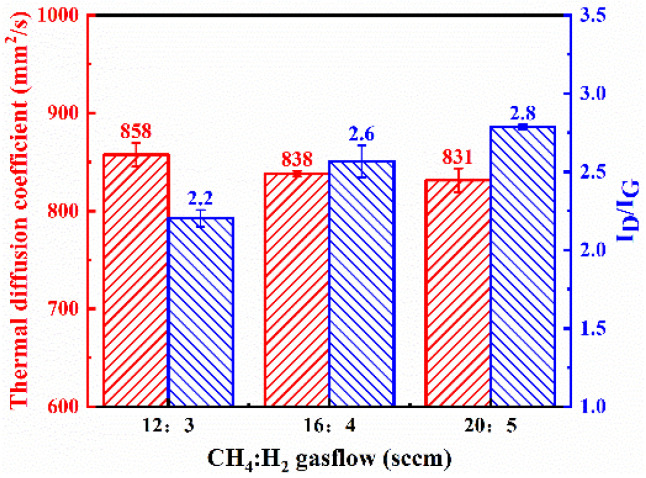
Thermal diffusion coefficient and *I*_D_/*I*_G_ ratio obtained from Raman spectroscopy of graphite film prepared under different CH_4_:H_2_ gas flow rates.

Under conditions with a catalyst concentration of 0.14 mol/L and without hydrogen plasma etching, while keeping other parameters consistent with the previous, the impact of growth gas flow rate on the deposition results was studied. The morphology of the graphite film under different process conditions is shown in Figure 4 and Figure 5.

As the gas flow rate increased, the deposited carbon nanosheets gradually became smoother, with a decrease in density and an increase in size. Their shapes and sizes are detailed in Table 1. As the growth gas flow rate increased further, the thermal diffusion coefficient of the modified graphite film decreased, and the defect density increased (Figure 3). Compared to increasing the catalyst concentration, increasing the growth gas flow rate had a more significant impact on the morphology and size of the carbon nanosheets. This is because, in addition to providing more carbon species, the gas flow rate directly affects the pressure inside the tubular furnace where the reaction occurs. Higher pressure significantly enhances the diffusion rate of active carbon species within the catalyst during deposition, accelerating the growth rate. Consequently, the carbon nanosheets tend to become smoother and larger in size.

#### 3.1.3. Hydrogen Plasma Etching Pretreatment

Under the process conditions of H_2_ gas flow rate of 15 sccm, catalyst concentration of 0.14 mol/L, gas flow rate of 20:5, and other parameters kept the same as previously described, the effect of hydrogen plasma pretreatment on the deposition results was studied.

**Figure 6:**
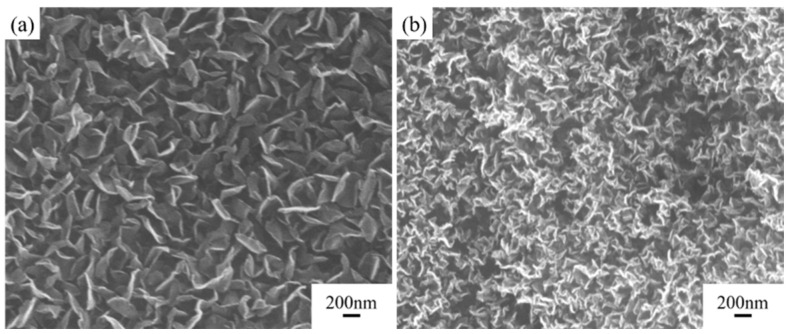
Comparison of structure and properties of carbon nanosheets pretreated with or without hydrogen plasma etching. SEM images of modified graphite film pretreated (**a**) without hydrogen plasma etching and (**b**) with hydrogen plasma etching.

As shown in Figure 6, hydrogen plasma etching significantly increases the density of carbon nanosheets, while also increasing the wrinkles in the nanosheets. This is partly because hydrogen plasma etching prevents catalyst annealing and agglomeration, thereby increasing catalyst density. Additionally, the etching process creates defects on the catalyst surface, introducing numerous nucleation sites, which leads to a noticeable increase in the density of carbon nanosheets.

The effectiveness of the pretreatment process depends on the etching temperature, as the etching process only takes effect when the temperature exceeds 450 °C. At lower temperatures, the reduction rate of the catalyst is relatively slow. Catalyst agglomeration mainly occurs in the temperature range near 500 °C. Therefore, only under high-temperature conditions can hydrogen plasma etching effectively control the structure and density of carbon nanosheets. In contrast, etching time has a minimal impact on the deposition results of the carbon nanosheets compared to the etching temperature.

**Figure 7:**
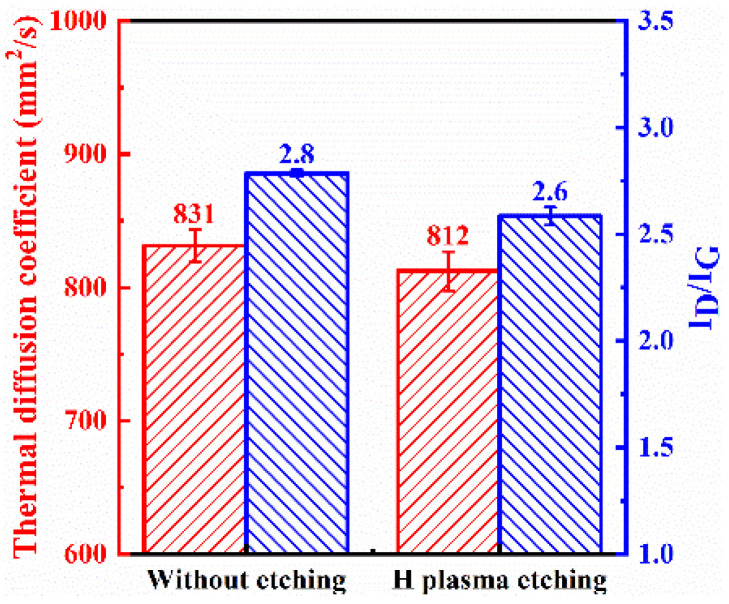
Thermal diffusion coefficient and *I*_D_/*I*_G_ ratio obtained from Raman spectroscopy of graphite film pretreated with or without hydrogen plasma etching.

The etching process not only affects the catalyst but also has some etching effect on the carbon of the graphite film, further increasing nucleation sites for carbon nanosheets and enhancing their density. However, this also disrupts the intact graphite structure, resulting in a slightly lower thermal diffusion coefficient for the modified graphite film prepared with the etching process, which decreases from 831.5 mm^2^/s to 812.4 mm^2^/s. Hydrogen plasma effectively removes amorphous carbon, so the final carbon nanosheets exhibit relatively low defect density, with the *I*_D_:*I*_G_ ratio decreasing from 2.8 to 2.6, which is shown in Figure 7.

In summary, the structure of carbon nanosheets is easy to control, with a broad process window. Increasing catalyst concentration, increasing growth gas flow rate, and hydrogen plasma etching all affect the size, morphology, and density of the carbon nanosheets.

Among these factors, the catalyst concentration has little effect on the size of the graphite film, but increasing the catalyst concentration can enhance the density of the carbon nanosheets on the surface of the graphite film, while decreasing the thermal diffusion coefficient of the modified graphite film and increasing the defect density. Increasing the growth gas flow rate leads to larger carbon nanosheet sizes and higher density, but also results in a decrease in the thermal diffusion coefficient and an increase in the defect density of the modified graphite film. High-temperature hydrogen plasma etching significantly increases the density of the carbon nanosheets, while also reducing the thermal diffusion coefficient of the modified graphite film.

During the deposition of carbon nanosheets, many factors influence the characteristics of the carbon nanosheets, such as gas composition, pressure, plasma power, reaction temperature, and reaction time, and interactions among these factors are often coupled. These factors affect the nucleation and growth processes of the carbon nanosheets, which caused the difference in the size and distribution of carbon nanosheets shown in Table 1. To better understand the growth behavior of carbon nanosheets, the PECVD deposition results were statistically analyzed and compared. The summarized results are presented in Table 2.

### 3.2. Carbon Nanosheets Growth Mechanism

The process of growing carbon nanosheets on the surface of the graphite film consists of three main stages: graphene nucleation, lateral graphene growth, and vertical graphene growth. The first two stages are identical to the lateral growth process of graphene [29]. It was found that during the nucleation and early growth stages of carbon nanosheets, almost no vertical carbon nanosheets were observed on the substrate. Instead, multi-layer graphene sheets, with a thickness of approximately 1–15 nm, grew in a parallel orientation to the surface of the substrate [30]. This process takes approximately 1–4 min. The initially formed graphene is thinner at the outer edges compared to the center. As the graphene reaches a sufficient size, the tips of its outer edges curl upward under the influence of the self-bias voltage generated by the plasma and intrinsic stress, leading to the formation of the initial carbon nanosheets which are perpendicular to the substrate.

The active carbon species have a very high surface diffusion rate in the plasma. Under the polarization effect at the tips of the carbon nanosheets, the diffusion rate of the active carbon species in the vertical direction is much higher than in the horizontal direction. As a result, the growth rate of the carbon nanosheets along the vertical direction is far greater than that in the horizontal direction. This leads to the growth of carbon nanosheets that are typically only a few to tens of atomic layers thick, with sharp edges [31].

The self-bias voltage generated by the plasma plays a crucial role in the growth of carbon nanosheets [32]. On the one hand, the electric field lowers the energy barrier at the edges of the initially formed graphene, allowing the carbon nanosheets to continue growing rather than merely nucleating. On the other hand, the presence of the electric field causes the carbon nanosheets to maintain a two-dimensional configuration, rather than forming closed surfaces.

In addition to the influence of the electric field, intrinsic stress is also considered a key driving factor in the vertical growth of carbon nanosheets. After the formation of horizontal graphene layers, tensile stress develops in the middle region of the nanosheets. This stress promotes upward bending, ultimately leading to the vertical orientation of the nanosheets relative to the substrate [33].

In addition to the self-bias voltage, PECVD offers advantages not available in other methods. Under the influence of high-energy electrons in the plasma, the precursor gases (methane and hydrogen) decompose into reactive species. Hydrogen radicals play a crucial role in the decomposition of methane by enhancing the conversion efficiency of methane and its radicals (CH_x_). Moreover, the etching effect of hydrogen effectively suppresses the formation of undesired carbon phases, thereby promoting the growth of large, continuous vertical graphene structures [20]. At the same temperature, the concentration of reactive carbon species in PECVD is higher than in other chemical vapor deposition methods. From another perspective, the preparation temperature for PECVD is much lower compared to other CVD methods [34]. In thermal chemical vapor deposition (CVD), materials are synthesized through the thermal decomposition of precursors that react on the substrate surface, typically requiring the substrate to be heated above the reaction temperature. In contrast, plasma-enhanced chemical vapor deposition (PECVD) generates a plasma state before the precursor reaches the substrate, enabling the reaction to proceed without the need for high substrate temperatures and making the process largely independent of substrate temperature [35]. PECVD can produce graphene on a substrate at temperatures as low as 400–600 °C, while other methods typically require at least 1000 °C. On the one hand, under such low temperature conditions, the graphite film maintains a high graphitization degree after treatment, avoiding the generation of excessive surface defects which could make it brittle and reduce its thermal conductivity. On the other hand, the lower temperature allows for a more controlled and gradual growth of carbon nanosheets, enabling better control over their size. In addition to the low temperature, the high-energy plasma impact also effectively reduces the amount of residual amorphous carbon on the surface, resulting in a high overall deposition quality.

From the experimental results in Section 3.1 showing a significant increase in the density of carbon nanosheets after hydrogen plasma etching, and that carbon nanosheets can still grow regardless of catalyst addition, it can be concluded that the mechanism for surface growth of carbon nanosheets follows the same principle described earlier. During the growth process, the hydrogen plasma etching on the surface of the graphite film generates defects. Active carbon species ionized from methane gas combine with these defects to nucleate. The graphene expands and grows outward from the nucleation sites. Therefore, the amount of nucleation is the key factor determining the density of the carbon nanosheets. In the process, factors such as increasing hydrogen plasma etching, surface oxidation, and catalyst addition all promote nucleation, introducing more nucleation sites, which ultimately leads to an increase in the density of the carbon nanosheets.

After nucleation, when the graphene reaches a certain size, it will begin to lift toward the surface under the influence of the plasma self-bias electric field, forming carbon nanosheets with a vertical orientation. Following this, the active carbon species diffuse along the carbon nanosheet more quickly. At this stage, the carbon nanosheets tend to grow along the sheet direction, rather than nucleating again on the original surface of the graphite film.

In this process, factors that increase carbon absorption and carbon content will result in larger carbon nanosheets. Therefore, increasing the gas flow rate (which increases the total carbon species content in the plasma) and adding catalysts (which increase the carbon absorption capacity of the graphite film surface) will both lead to the formation of larger carbon nanosheets.

### 3.3. GF/Al Composites

The graphite films prepared using three different processes (without catalyst, with catalyst but without etching, and with catalyst and etching) were used to fabricate the graphite/aluminum composites, which were denoted as CNS-1, CNS-2, and CNS-3. The three groups of processes and their selected conditions are as follows: CNS-1: Graphite films without catalyst treatment, featuring the lowest surface carbon nanosheet density. The growth gas flow was CH_4_:H_2_ = 16:4. CNS-2: Based on CNS-1 graphite films, further treated with a catalyst at a concentration of 0.07 mol/L, resulting in an increased carbon nanosheet density and reduced nanosheet size. CNS-3: Produced under conditions that achieved the highest carbon nanosheet density, involving hydrogen plasma etching, a catalyst concentration of 0.14 mol/L, and a growth gas flow of 20:5. Under these conditions, the carbon nanosheet density increased significantly, and the nanosheet size became markedly smaller. The volume fraction of the composites is 42%, and the thickness of the composites is 3 mm.

#### 3.3.1. Microstructure and Elemental Distribution of GF/Al Composites

**Figure 8:**
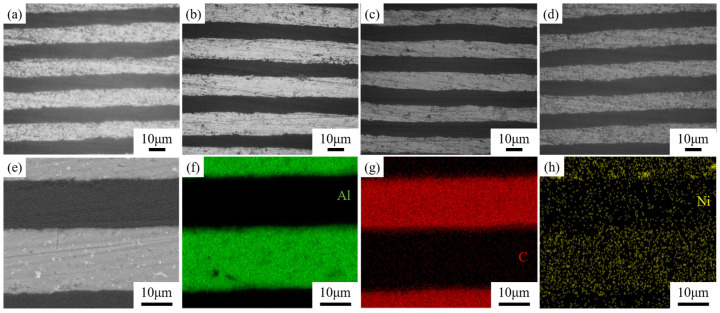
Metallographic structure and SEM image of GF/Al composites with surface-modified carbon nanosheets prepared under different processes. Metallographic structure of (**a**) GF/Al, (**b**) CNS-1, (**c**) CNS-2, (**d**) CNS-3; (**e**) SEM image of CNS-2; EDS mapping of (**f**) Al, (**g**) C, and (**h**) Ni.

**Figure 9:**
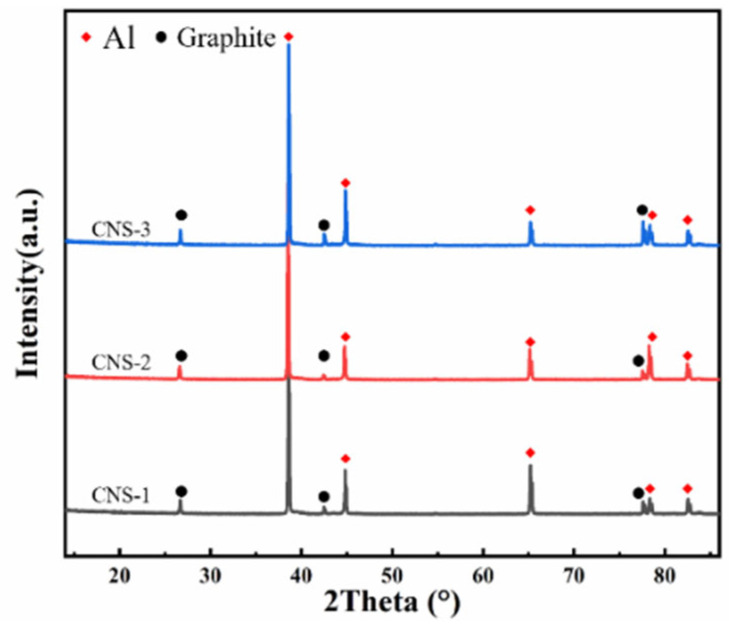
XRD pattern of GF/Al composites modified with carbon nanosheets using different processes.

Figure 8a–d shows the metallographic structure of GF/Al composites with surface-modified carbon nanosheets prepared under different processes. The dark areas represent the modified graphite film, while the light areas are the aluminum matrix. It can be observed that the layer spacing of all four composites is relatively uniform, with good interfacial bonding and no signs of voids or interfacial debonding. Due to the presence of alloying elements, some fine precipitates can be observed. There are no significant differences in the metallographic structures of GF/Al composites modified with different carbon nanosheets. Figure 9 shows the XRD pattern of GF/Al composites modified with carbon nanosheets using different processes. It can be seen that only the characteristic peaks of the aluminum matrix and graphite film are detected in the three types of carbon nanosheet-modified GF/Al composites. No characteristic peaks of precipitates or interfacial reaction products are observed, indicating that the content of precipitates and interfacial reaction products is relatively low.

#### 3.3.2. Thermal Performance of GF/Al Composites

**Figure 10:**
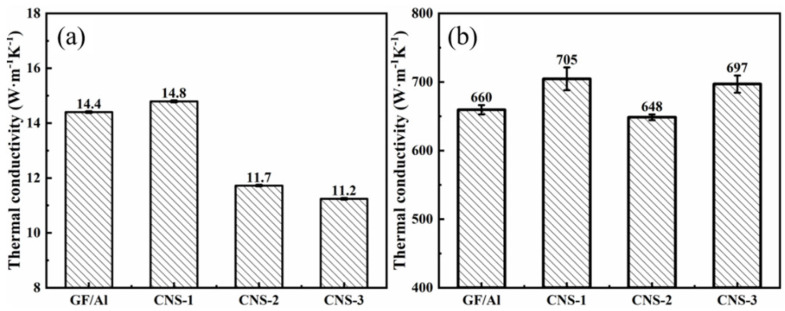
Thermal conductivity of GF/Al composites modified with carbon nanosheets using different modification processes in (**a**) z-direction; (**b**) xy-direction.

Figure 10 shows the thermal conductivity of GF/Al composites modified with carbon nanosheets using different modification processes. CNS-1 has the highest thermal conductivity, with its thermal conductivity in the z-direction being close to that of GF/Al, and its thermal conductivity in the xy-direction increasing from 659.5 W/(m·K) to 704.6 W/(m·K). The addition of carbon nanosheets improves the interfacial bonding within the composite material, thereby enhancing the overall thermal conductivity of the composite.

When the catalyst is added, the z-direction thermal conductivity of CNS-2 and CNS-3 is lower than that of GF/Al, and the xy-direction thermal conductivity of CNS-3 is also lower than that of GF/Al. The decrease in the composite’s thermal conductivity may be due to the residue of the catalyst. The addition of Ni catalyst further reduces the thermal conductivity of aluminum, and because of the different heat transfer paths, this reduction in the aluminum matrix has a greater impact on z-direction thermal conductivity than on xy-direction thermal conductivity. Figure 8e–h shows the energy spectrum of the CNS-2 composite, where Ni catalyst residue and its diffusion into the aluminum matrix can be observed.

As mentioned earlier, during the preparation of the composite, pressure must be applied perpendicular to the graphite film direction in both the preform fabrication and pressure infiltration processes. Under this pressure, the carbon nanosheets tend to lie down onto the graphite film rather than remaining vertically attached to its surface. These flattened carbon nanosheets share the same anisotropic thermal conductivity characteristics as the graphite film, with poorer thermal conductivity in the direction perpendicular to the carbon nanosheets. Consequently, the flattened carbon nanosheets increase thermal resistance in the z-direction. Therefore, it can be observed that from the CNS-2 process to the CNS-3 process, as the density of carbon nanosheets increases, the z-direction thermal conductivity of the composite further decreases.

Although the introduction of the catalyst reduces the thermal conductivity of the aluminum matrix, the matrix’s contribution to the overall thermal conductivity in the xy-direction is minimal due to the different heat transfer paths. Thus, in the CNS-2 process, the xy-direction thermal conductivity of the composite still improves. After modification with the CNS-2 process, the thermal conductivity of the GF/Al composite slightly decreases from 660 W/(m·K) to 649 W/(m·K). However, after modification with the CNS-3 process, the thermal conductivity of the GF/Al composite increases from 660 W/(m·K) to 697 W/(m·K).

## 4. Conclusions

In summary, the effects of catalyst addition and concentration, growth gas flow rate, and hydrogen plasma pretreatment on the size, morphology, and density of deposited carbon nanosheets in RF-PECVD were studied. The effect of fabrication processes on the thermal conductivity of GF/Al composites was investigated. The main conclusions are as follows:

(1) The catalyst concentration had minimal impact on the size of the graphite film, but increasing the concentration enhanced the density of carbon nanosheets on the film surface, while also reducing the thermal diffusivity and increasing the defect density of the modified graphite film. Increasing the growth gas flow rate resulted in larger and denser carbon nanosheets, which also led to a decrease in the thermal diffusivity and an increase in defect density. High-temperature hydrogen plasma etching significantly increased the density of carbon nanosheets but decreased in the thermal diffusivity of the modified graphite film.
(2) The experimental results indicate that the nucleation and growth processes are the key factors affecting the density and size of carbon nanosheets, respectively. Increasing hydrogen plasma etching, surface oxidation, and catalyst addition all promote nucleation by introducing nucleation sites, leading to a higher density of carbon nanosheets. Increasing the gas flow rate raises the total carbon species content in the plasma, and catalyst addition increases carbon absorption on the graphite film surface, both of which ultimately increase the size of the carbon nanosheets.
(3) Graphite film/aluminum composites were prepared using the pressure infiltration method, demonstrating good bonding. Among the conditions, the composites prepared without a catalyst showed the highest thermal conductivity, with an xy-direction thermal conductivity of 705 W/(m·K) and a z-direction thermal conductivity of 14.8 W/(m·K).

## Figures and Tables

**Table 1:** Size and distribution of carbon nanosheets under different process conditions.

Catalyst Concentration (mol/L)	Growth Gas Flow CH_4_:H_2_ (sccm)	Hydrogen Plasma Etching	Carbon Nanosheets Length (nm)	Density of Carbon Nanosheets (/μm^2^)	Thickness of Synthesized Films (nm)
-	16:4	-	344.7	13.9	731
0.07	16:4	-	243.8	18.9	736
0.10	16:4	-	411.5	17.2	718
0.14	16:4	-	499.8	17.5	647
0.14	12:3	-	400.5	26.7	660
0.14	16:4	-	466.5	17.1	670
0.14	20:5	-	546.7	14.7	724
0.14	20:5	-	546.7	14.7	724
0.14	20:5	650 °C	131.7	71.6	375

**Table 2:** Summary table of carbon nanosheet deposition results by PECVD.

Carbon Source/Carrier Gas	Substrate	Main Condition	Density of Carbon Nanosheets * (/μm^2^)	Carbon Nanosheets Length * (nm)	Application Field	Ref.
CH_4_/Ar H_2_	Cu mesh	DC-PECVD P: 150 W p: 6 mbar T: 600 °C	320	96.7	Hydrophobic coating/electrodes of fuel cells	[23]
CH_4_/H_2_	SiO_2_	MPECVD P: 1300 W p: 0.5 torr T: 600 °C	12	465	Room temperature gas sensor	[24]
Essential oil/H_2_	Si/Quartz	RF-PECVD P: 500 W p: 0.2 mbar T: 700 °C	48	220	Antibacterial/cytotoxicity	[25]
Ethanol	Soda-lime glass	RF-PECVD P: 300 W p: 6 mbar T: 650 °C	38	175	Thermochromic applications	[26]
CH_4_/Ar H_2_	Si	CCPPECVD P: 300 W p: 0.25 torr T: 750 °C	127	116	-	[27]
CH_4_/Ar	Si	RF-PECVD P: 150 W p: 5 × 10^−3^ mbar T: 800 °C	103	131		[19]
CH_4_/Ar H_2_	Pt	RF-PECVD P: 550 W p: 22 mTorr T: 720 °C	4–5	734	Polymer electrolyte fuel cell	[28]
CH_4_/H_2_	Graphite film	MPECVD P: 300 W p: 68–100 Pa T: 650 °C	13.9–71.6	131.7–546.7	Thermally conductive reinforcement for Al matrix composites	Present study

* Data were obtained by analyzing SEM images using ImageJ software1.53t.

## Data Availability

The data are available from the corresponding author on reasonable request.
